# Cardiac MRI quantitative tissue characterization of right atrial mass using mDixon and parametric mapping

**DOI:** 10.1007/s00392-017-1129-7

**Published:** 2017-06-19

**Authors:** Tomas Lapinskas, Marc Kouwenhoven, Bernhard Schnackenburg, Tamar Bigvava, Katharina Wassilew, Rolf Gebker, Stephan Jacobs, Remigijus Zaliunas, Burkert Pieske, Sebastian Kelle

**Affiliations:** 1 Department of Internal Medicine/Cardiology, Deutsches Herzzentrum Berlin, Augustenburger Platz 1, 13353 Berlin, Germany; 2 Department of Cardiology, Medical Academy, Lithuanian University of Health Sciences, Kaunas, Lithuania; 3 Philips Healthcare, Best, The Netherlands; 4 Philips Healthcare, Hamburg, Germany; 5 Tbilisi Heart and Vascular Clinic, Tbilisi, Georgia; 6 Department of Cardiovascular Pathology, Cardiothoracic and Vascular Surgery, Deutsches Herzzentrum Berlin, Berlin, Germany; 7 Department Cardiothoracic and Vascular Surgery, Deutsches Herzzentrum Berlin, Berlin, Germany; 8 DZHK (German Centre for Cardiovascular Research), Partner Site Berlin, Berlin, Germany

Sirs:

A 49-year-old man presented with episodes of dizziness during head and neck movements and increased fatigue. There was no associated chest pain, dyspnea, orthopnea, paroxysmal nocturnal dyspnea, palpitations, nausea, vomiting or limb swelling. His medical and family history was unremarkable and physical examination was normal. Transthoracic echocardiography (TTE) (Fig. 1a) showed a large 42 × 25 mm and slightly mobile mass in the right atrium (RA) attached to the interatrial septum (IAS). Transesophageal echocardiography (TEE) (Fig. 1b) demonstrated partial obstruction of the superior vena cava.

**Fig. 1:**
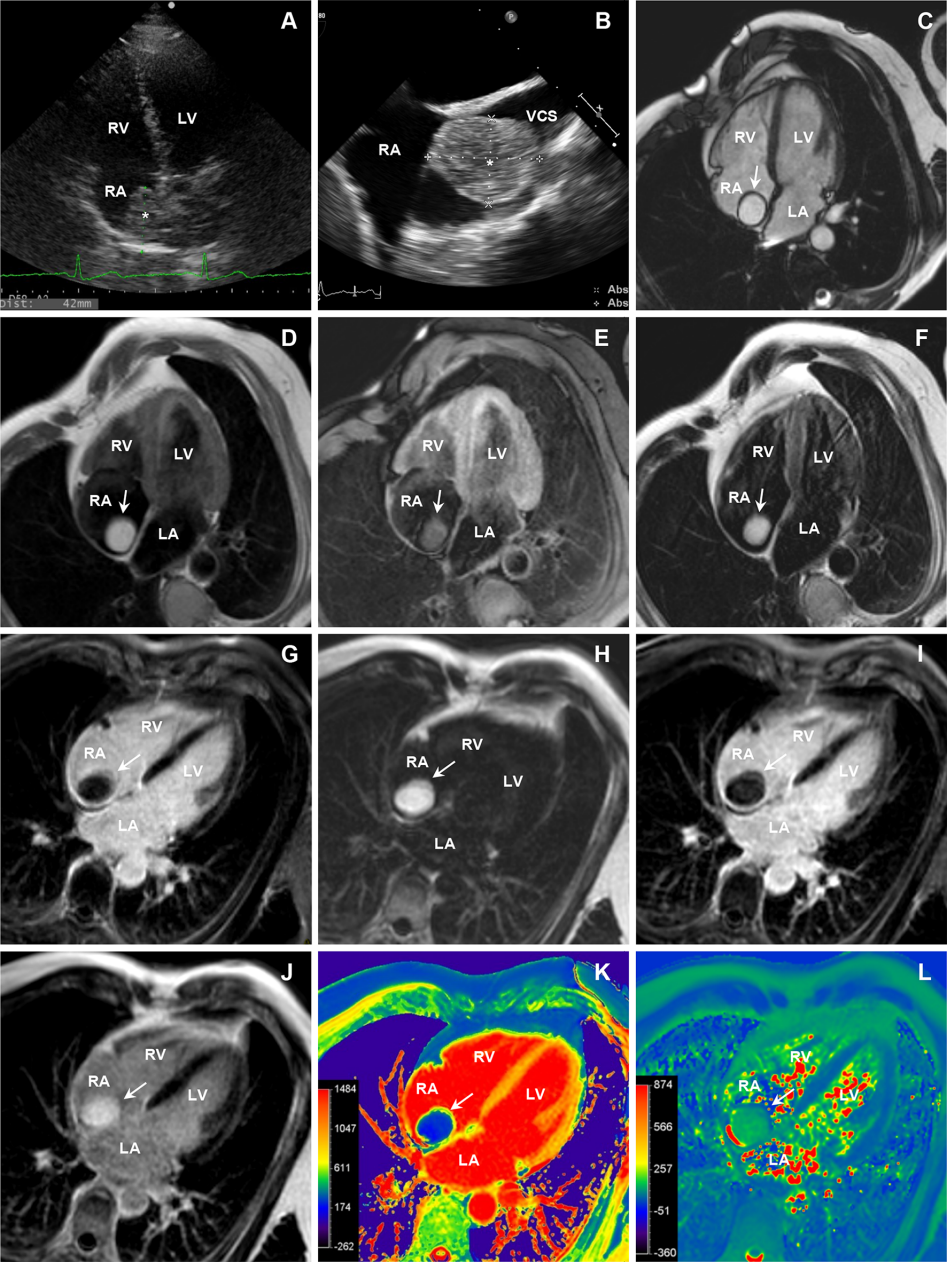
TTE (**a**) and TEE (**b**) show large, slightly mobile mass in RA (*asterisk*) attached to the IAS. CMR cine imaging in four-chamber (**c**) view demonstrates mass with oval shape and regular borders (*arrow*) in RA adherent to IAS. T1-weighted spin-echo without (**d**) and with (**e**) fat saturation sequences. Tumor (*arrow*) is hyperintense in T1-weighted spin-echo sequence without fat saturation and hypointense after fat suppression prepulses. On T2-weighted spin-echo (**f**) sequence the mass (*arrow*) appears isointense. On LGE (**g**) imaging, the mass (*arrow*) did not enhance. Single breath-hold ECG-gated multi-echo chemical shift-based (mDIXON) sequence was used to generate separate fat-only (**h**), water-only (**i**) and in-phase images (**j**). The mass demonstrated high signal intensity on fat-only and in-phase and low signal intensity on water-only images. Native T1-mapping (**k**) shows significantly lower T1 values of the tumor compared with normal myocardium, but similar to subcutaneous fat. Pre-contrast T2-mapping (**l**) displays higher values than normal myocardium. *LV* left ventricle, *LA* left atrium, *RV* right ventricle, *RA* right atrium, *IAS* interatrial septum, *VCS* vena cava superior, *TTE* transthoracic echocardiography, *TEE* transesophageal echocardiography, *CMR* cardiac magnetic resonance, *LGE* late gadolinium enhancement, *ECG* electrocardiography. *Asterisk* and *arrow* indicate cardiac mass

Cardiac magnetic resonance (CMR) was performed to determine specific mass characteristics using a 1.5-Tesla Philips Achieva (Philips Healthcare, Best, The Netherlands) scanner with a 32-channel cardiac surface coil. Cine images (Fig. 1c) showed a homogeneous 32 × 27 × 49 mm oval-shaped tumor with regular borders in the RA adherent to the IAS. The mass appeared hyperintense on non-contrast T1-weighted spin-echo images (Fig. 1d) and became hypointense after application of fat suppression prepulses (Fig. 1e). On T2-weighted spin-echo sequence (Fig. 1f) the mass appeared isointense. The tumor was poorly perfused during first-pass perfusion imaging and did not enhance after administration of full dose of contrast agent (Fig. 1g).

Single breath-hold three-dimensional (3D) ECG-gated multi-echo chemical shift-based (mDixon) sequence was used for advanced tissue characterization. In addition, pre-contrast (native) T1 and T2 relaxation times and post-contrast T1 relaxation times were calculated from single breath-hold two-dimensional (2D) modified Look–Locker inversion recovery (MOLLI) sequence [1].

The mass had high signal intensity on fat-only (Fig. 1h) and low signal intensity on water-only (Fig. 1i) mDixon images. In the in-phase (Fig. 1j) mDixon images the mass appeared hyperintensive. Moreover, native T1-mapping (Fig. 1k) showed homogeneous and significantly lower T1 values (274 ms) for the tumor compared with the normal myocardium (1013 ms). The T1 values of the tumor were similar to the T1 of subcutaneous adipose tissue (289 ms). The extracellular volume fraction (ECV) of the mass was lower than that of the myocardium (17.8 vs. 32.4%, respectively). Pre-contrast T2-mapping (Fig. 1l) showed higher values for the tumor (133 ms) than for normal myocardium (56 ms). Following these advanced tissue characterization findings, the cardiac mass was diagnosed as a benign lipoma. The diagnosis was confirmed after surgery and histological evaluation (Fig. 2).

**Fig. 2:**
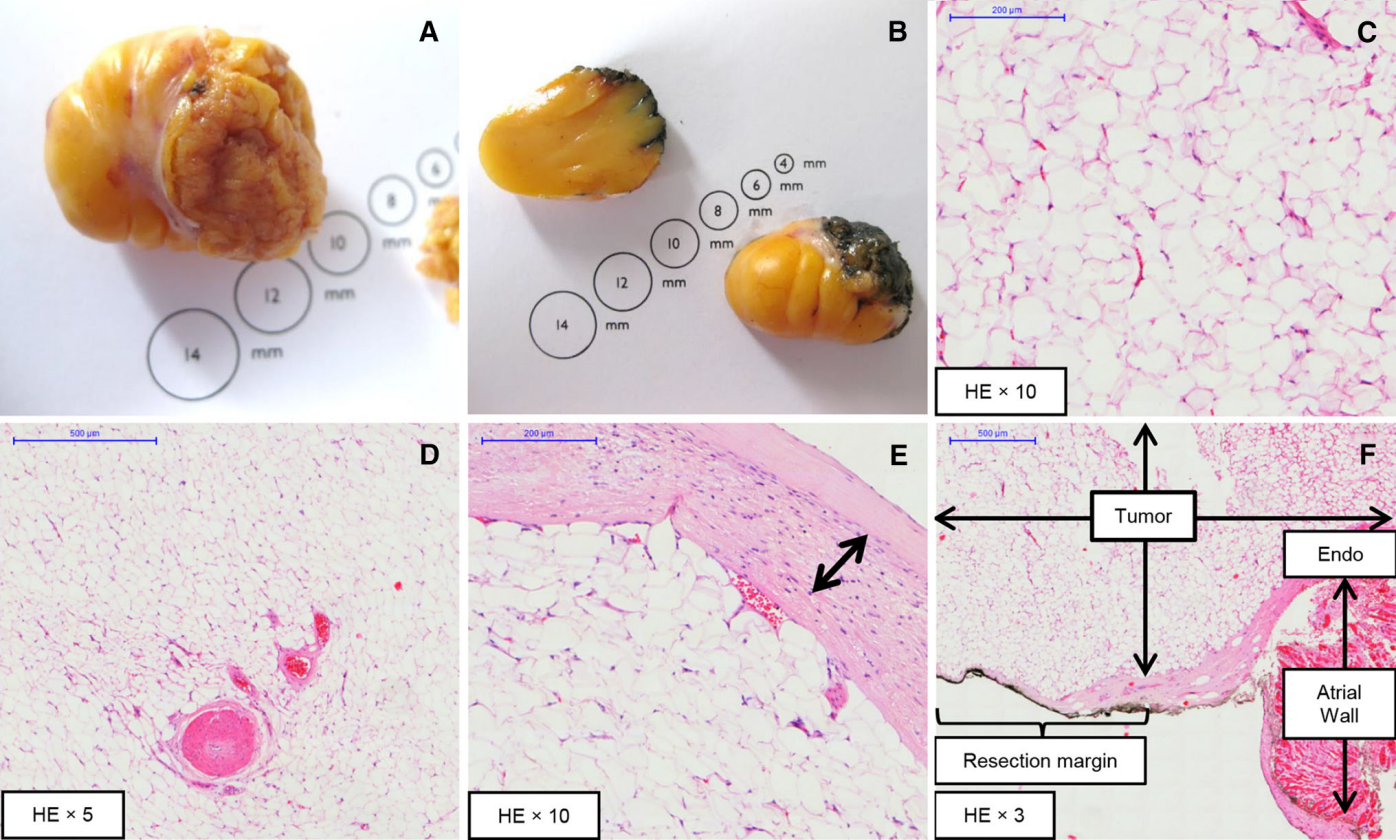
Photograph of whole (**a**) and sectioned (**b**) RA mass after surgical excision. Hematoxylin and eosin staining depicts the lipomatous tumor composed of mature adipocytes without nuclear atypia (**c**) and numerous capillary-sized vessels and vessels with thick muscular walls (**d**). Tumor is covered by a fibrous capsule (**e**) which contains bundles of chronic inflammatory cells and bundles of cells with myofibroblastic appearance (**f**). The tumor is attached to the endocardium of the RA and there is no evidence of an infiltrative growth pattern into the atrial wall (**f**). The tumor reaches the resection margin (*inked black*)

The diagnosis of cardiac masses is frequently challenging. Primary cardiac tumors are very rare [2] compared to metastatic tumors of the heart, which are at least 20 times more common [3].

Cardiac lipomas are benign tumors which are typically asymptomatic and found incidentally. The presence of symptoms depends upon the size and location of the tumor. They can cause heart failure, arrhythmias, embolization and obstructive symptoms due to blockage of vena cava or by hindering the opening and closing of the valves.

Echocardiography remains the first-choice imaging modality, providing high sensitivity in detecting cardiac masses, particularly by the transesophageal approach. The differential diagnosis of cardiac masses using echocardiography is limited. Some speculations can be made by assessing tumor’s location, size, attachment, mobility, echogenicity or calcification. However, all these findings are nonspecific. In our case the differential diagnosis of detected mass included benign and malignant tumors. Benign masses within the atria encompassed thrombus, myxoma, rhabdomyoma, fibroma, and fibroelastoma, as well as lipoma.

CMR offers distinct advantages, including 3D and multiplanar images, a large field-of-view, excellent contrast resolution with high spatial resolution and the potential to characterize specific tissues based on their signal intensity. Conventionally tumors content in fat and in water can be assessed by T1-weighted and T2-weighted imaging. Late gadolinium enhancement (LGE) strengthens CMR value as it provides complementary tissue characterization [4]. However, routine CMR protocols ensure only visual assessment of the tissues and new quantitative methods are needed while their validation is still lacking.

Recently established fat–water separated (mDixon) imaging and quantitative parametric (T1 and T2) mapping techniques could expand CMR imaging repertoire for the differentiation of cardiac masses on the basis of tissue characterization. These techniques demonstrate unique ability to achieve noninvasive in vivo characterization of cardiac tumors preoperatively [5].

One of the principal roles of noninvasive cardiac imaging is to determine whether a mass is benign or malignant since the diagnosis may immediately change the prognosis and management. Standard CMR protocols for cardiac masses include black-blood T1-weighted spin-echo sequences before and after injection of gadolinium with and without fat suppression, black-blood T2-weighted spin-echo imaging and early and LGE [6]. Fundamentally, the principal intention of these CMR techniques is to generate an image where fat tissue appears significantly brighter (or hyperintense) in contrast to non-adipose tissue. Unfortunately, these imaging techniques have a variety of limitations: high-intensity slow flow artifacts at the subendocardial surface impede accurate evaluation and long acquisition time may lead to spatial misregistration [7]. Furthermore, conventional CMR techniques rely on the visual assessment of signal intensity of tissues and observer’s experience.

More detailed determination of tumor-specific features using CMR is required and advanced noninvasive tissue characterization may become an option. A method of fat and water separation based on proton chemical shift imaging was first described by Dixon [8]. Early research papers describing fat–water separated cardiac imaging were published in 2005 [9] with following publications in 2009 [10]. Dixon showed that separate fat and water images can be generated by selecting a number of appropriate echoes or points that they sample. Eggers et al. explored a two-point method for fat and water imaging and concluded that this technique ensures higher spatial resolution and better signal-to-noise ratio and may reduce scan time [11]. Fat suppression is frequently an integral part of the study and the mDixon protocol demonstrates a potential to replace conventional fat-suppression techniques. Fat–water separated imaging provides more reliable fat detection on fat-only images, whereas water-only images enable the identification of edematous lesions or effusions [12]. The essential advantage of the current fat–water separated imaging technique is the ability to measure total fat volume in a 3D approach by segmentation of voxels that predominantly contain adipose cells [13]. Currently, all known CMR vendors have implemented fat–water separated cardiac imaging sequences at least for research purposes.

New quantitative myocardial mapping techniques quantify within each pixel the T1 and/or T2 relaxation times that can be displayed as color maps to facilitate visual interpretation. All tissues exhibit intrinsic T1 relaxation times which are determined by the composition of the cellular and intersticial components [14]. The longitudinal relaxation T1 time is increased with myocardial edema, fibrosis or deposition of amyloid and is reduced in lipid accumulation, Anderson–Fabry disease or iron overload. Furthermore, T1 mapping after injection of contrast agent in conjunction with the pre-contrast (native) T1 mapping and hematocrit value enables calculation of ECV which is a measure of the proportion of extracellular space within the myocardium [15]. Mapping sequences allowed significant improvement in tissue characterization and may become a new tool for noninvasive differential diagnosis of cardiac tumors [5]. Moreover, native T1 and T2 mapping techniques do not require injection of gadolinium-based contrast agent and can be used in patients with severe renal dysfunction.

Ferreira et al. were the first to demonstrate the feasibility of pre-contrast T1 mapping for quantitative characterization of cardiac mass to confirm clinical diagnosis [16]. It was demonstrated that technique is more sensitive to detect hidden myocardial injury and is less prone to artifacts compared with T2-weighted imaging and even T2 mapping [17].

The implementation of T2-weighted imaging has enabled to visualize increased myocardial water content. Importantly, these sequences are essential to establish the correct diagnosis of myocardial inflammation, especially when the LGE is absent [18]. However, this technique is very sensitive to the various artifacts, induced by irregular heart rate or inabilities to maintain breath-hold. In addition, T2-weighted sequences are unable to detect generalized myocardial edema [19]. The quantitative T2 mapping overcomes these limitations. To generate T2 map, a bSSFP sequence is applied to acquire three single-shot T2-weighted images [20]. There are only few publications describing cardiac tumors assessed by parametric mapping. Recent study conducted by Caspar et al. demonstrated that the value of native T2 is short for calcifications, intermediate for the melanoma and very long for lipomas [5].

Extensive movement of the tumor may influence the applicability of the parametric mapping and may degrade the accuracy of the technique [21]. In both T1 and T2 mapping measurements the in-plane displacement of the tumor estimated in the original images between the echoes/delays was less that 1 mm. We also do not expect large effects from through-plane motion as we sampled data only in diastole for 170 ms (T1) and 75 ms (T2).

Preoperative diagnostic workup should include these new imaging modalities, alongside the more conventional ones such as echocardiography. The CMR can provide additional valuable information that cannot be obtained by other imaging techniques. Furthermore, this information may even be helpful for the pathologist in controversial cases.

With this case, we demonstrate the additional value of current CMR techniques to noninvasively characterize cardiac masses, especially when standard imaging protocols are challenged. Fat–water separation (mDixon) and quantitative parametric mapping techniques could be suitable for differential diagnosis of cardiac tumors preoperatively. However, the technique lacks histological validation in larger clinical trials.

## References

[1] Messroghli DR, Radjenovic A, Kozerke S, Higgins DM, Sivananthan MU, Ridgway JP (2004). Modified look-locker inversion recovery (MOLLI) for high-resolution T1 mapping of the heart. Magn Reson Med.

[2] Reynen K (1996). Frequency of primary tumors of the heart. Am J Cardiol.

[3] Lam KY, Dickens P, Chan AC (1993). Tumors of the heart. A 20-year experience with a review of 12,485 consecutive autopsies. Arch Pathol Lab Med.

[4] Motwani M, Kidambi A, Herzog BA, Uddin A, Greenwood JP, Plein S (2013). MR imaging of cardiac tumors and masses: a review of methods and clinical applications. Radiology.

[5] Caspar T, El Ghannudi S, Ohana M, Labani A, Lawson A, Ohlmann P, Morel O, De Mathelin M, Roy C, Gangi A, Germain P (2017). Magnetic resonance evaluation of cardiac thrombi and masses by T1 and T2 mapping: an observational study. Int J Cardiovasc Imaging.

[6] Kramer CM, Barkhausen J, Flamm SD, Kim RJ, Nagel E, Society for Cardiovascular Magnetic Resonance Board of Trustees Task Force on Standardized Protocols (2013). Standardized cardiovascular magnetic resonance (CMR) protocols 2013 update. J Cardiovasc Magn Reson.

[7] Farrelly C, Shah S, Davarpanah A, Keeling AN, Carr JC (2012). ECG-gated multiecho Dixon fat-water separation in cardiac MRI: advantages over conventional fat-saturated imaging. AJR.

[8] Dixon WT (1984). Simple proton spectroscopic imaging. Radiology.

[9] Reeder SB, Marki M, Yu H, Hellinger JC, Herfkens RJ, Pelc NJ (2005). Cardiac CINE imaging with IDEAL water-fat separation and steady-state free precession. J Magn Reson Imaging.

[10] Kellman P, Hernando D, Shah S, Zuehisdorff S, Jerecic R, Mancini C, Liang ZP, Arai AE (2009). Multiecho Dixon fat and water separation method for detecting fibrofatty infiltration in the myocardium. Magn Reson Med.

[11] Eggers H, Brendel B, Duijndam A, Herigault G (2011). Dual-echo Dixon imaging with flexible choice of echo times. Magn Reson Med.

[12] Ma J (2008). Dixon techniques for water and fat imaging. J Magn Reson Imaging.

[13] Homsi R, Meier-Schroers M, Gieseke J, Dabir D, Luetkens JA, Kuetting DL, Naehle CP, Marx C, Schild HH, Thomas DK, Sprinkart AM (2016). 3D-Dixon MRI based volumetry of peri- and epicardial fat. Int J Cardiovasc Imaging.

[14] Coelho-Filho OR, Shah RV, Mitchell R, Neilan TG, Moreno H, Simonson B, Kwong R, Rosenzweig A, Das S, Jerosch-Herold M (2013). Quantification of cardiomyocyte hypertrophy by cardiac magnetic resonance: implications for early cardiac remodeling. Circulation.

[15] Moon JC, Messroghli DR, Kellman P, Piechnik SK, Robson MD, Ugander M, Gatehouse PD, Arai AE, Friedrich MG, Neubauer S, Schulz-Menger J, Schelbert EB, Society for Cardiovascular Magnetic Resonance Imaging, Cardiovascular Magnetic Resonance Working Group of the European Society of Cardiology (2013). Myocardial T1 mapping and extracellular volume quantification: a Society for Cardiovascular Magnetic Resonance (SCMR) and CMR Working Group of the European Society of Cardiology consensus statement. J Cardiovasc Magn Reson.

[16] Ferreira VM, Holloway CJ, Piechnik SK, Karamitsos TD, Neubauer S (2013). Is it really fat? Ask a T1-map. Eur Heart J Cardiovasc Imaging.

[17] Radunski UK, Lund GK, Säring D, Bohnen S, Stehning C, Schnackenburg B, Avanesov M, Tahir E, Adam G, Blankenberg S, Muellerleile K (2017). T1 and T2 mapping cardiovascular magnetic resonance imaging techniques reveal unapparent myocardial injury in patients with myocarditis. Clin Res Cardiol.

[18] Friedrich MG, Sechtem U, Schulz-Menger J, Holmvang G, Alakija P, Cooper LT, White JA, Abdel-Aty H, Gutberlet M, Prasad S, Aletras A, Laissy JP, Paterson I, Filipchuk NG, Kumar A, Pauschinger M, Liu P, International Consensus Group on Cardiovascular Magnetic Resonance in Myocarditis (2009). Cardiovascular magnetic resonance in myocarditis: a JACC White Paper. J Am Coll Cardiol.

[19] Palazón RJP, Arqués MS, González SP, de Caralt Robira TM, López MTC, Pérez JTO (2015). Parametric methods for characterizing myocardial tissue by magnetic resonance imaging (part 2): T2 mapping. Radiologia.

[20] Kellman P, Aletras AH, Mancini C, McVeigh ER, Arai AE (2007). T2-prepared SSFP improves diagnostic confidence in edema imaging in acute myocardial infarction compared to turbo spin echo. Magn Reson Med.

[21] Xue H, Shah S, Greiser A, Guetter C, Littmann A, Jolly MP, Arai AE, Zuehlsdorff S, Guehring J, Kellman P (2012). Motion correction for myocardial T1 mapping using image registration with synthetic image estimation. Magn Reson Med.

